# Tetramerization of SAMHD1 Is Required for Biological Activity and Inhibition of HIV Infection*[Fn FN2]

**DOI:** 10.1074/jbc.M112.443796

**Published:** 2013-02-20

**Authors:** Junpeng Yan, Sarabpreet Kaur, Maria DeLucia, Caili Hao, Jennifer Mehrens, Chuanping Wang, Marcin Golczak, Krzysztof Palczewski, Angela M. Gronenborn, Jinwoo Ahn, Jacek Skowronski

**Affiliations:** From the ‡ Departments of Molecular Biology and Microbiology and; ‖ Pharmacology, Case Western Reserve School of Medicine, Cleveland, Ohio 44106 and; the § Department of Structural Biology and; ¶ Pittsburgh Center for HIV Protein Interactions, University of Pittsburgh School of Medicine, Pittsburgh, Pennsylvania 15260

**Keywords:** HIV, Innate Immunity, Protein Chemistry, Protein Complexes, Virus

## Abstract

SAMHD1 is a dGTP-activated dNTPase that has been implicated as a modulator of the innate immune response. In monocytes and their differentiated derivatives, as well as in quiescent cells, SAMHD1 strongly inhibits HIV-1 infection and, to a lesser extent, HIV-2 and simian immunodeficiency virus (SIV) because of their virion-associated virulence factor Vpx, which directs SAMHD1 for proteasomal degradation. Here, we used a combination of biochemical and virologic approaches to gain insights into the functional organization of human SAMHD1. We found that the catalytically active recombinant dNTPase is a dGTP-induced tetramer. Chemical cross-linking studies revealed SAMHD1 tetramers in human monocytic cells, in which it strongly restricts HIV-1 infection. The propensity of SAMHD1 to maintain the tetrameric state *in vitro* is regulated by its C terminus, located outside of the catalytic domain. Accordingly, we show that the C terminus is required for the full ability of SAMHD1 to deplete dNTP pools and to inhibit HIV-1 infection in U937 monocytes. Interestingly, the human SAMHD1 C terminus contains a docking site for HIV-2/SIVmac Vpx and is known to have evolved under positive selection. This evidence indicates that Vpx targets a functionally important element in SAMHD1. Together, our findings imply that SAMHD1 tetramers are the biologically active form of this dNTPase and provide new insights into the functional organization of SAMHD1.

## Teaser

**Background:** SAMHD1, a dGTP-activated dNTPase, inhibits retrovirus infection at the reverse transcription step in monocytes and quiescent T lymphocytes.

**Results:** dGTP-induced SAMHD1 tetramerization correlates with its functional activation.

**Significance:** Learning how SAMHD1 function is regulated is important for understanding innate and anti-viral immunity.

## Introduction

Human immunodeficiency virus type I is relatively inefficient at infecting cells with key innate immunity roles, such as monocytes, dendritic cells, and macrophages, and this may contribute to the poor innate response to HIV infection (1–3). Dendritic cells are largely refractory to HIV-1 infection (4), and monocytes and macrophages, despite their susceptibility to infection, do not provide a conducive environment for HIV-1 cDNA synthesis. As a result, infection is inhibited at early post-entry steps (reviewed in 5). The finding that a virion-associated, accessory virulence factor, Vpx, encoded by HIV-2 and related simian immunodeficiency viruses (SIV),^3^ is required for efficient transduction of dendritic cells and macrophages revealed a restrictive mechanism against these viruses; the subsequent discovery that Vpx also relieves the inhibition of HIV-1 infection in restricting cell types demonstrated that the inhibitory mechanism broadly targets primate retroviruses (6–11). These findings together set the stage for identification of SAMHD1 (sterile α motif domain- and HD domain-containing protein 1) as the cellular anti-viral factor responsible for the early post-entry inhibition of HIV/SIV infection in monocyte-derived cell lineages and in quiescent CD4^+^ lymphocytes (12–16).

SAMHD1 is a nuclear protein possessing deoxyribonucleoside triphosphate triphosphohydrolase (dNTPase) activity. Its dNTPase activity is stimulated by deoxyguanosine triphosphate (dGTP) binding at a predicted allosteric site (17, 18). The protein comprises a conserved, N-terminal sterile α motif (SAM) domain, which is preceded by a nuclear localization signal (19, 20), a catalytic HD domain, and a C-terminal region that is divergent among SAMHD1 proteins from different primate and vertebrate species (21, 22). A crystal structure of a SAMHD1 fragment comprising catalytic core reveals that the protein is dimeric (17). Experimental evidence supports a model in which SAMHD1 inhibits primate lentivirus infection in quiescent cells by depleting dNTP levels below the threshold required for viral reverse transcriptase to efficiently synthesize proviral cDNA (23, 24).

Vpx counteracts SAMHD1 by programming it for degradation by the proteasome (12, 13). In particular, SIVmac Vpx recognizes a specific determinant in the C terminus of human SAMHD1 and loads it onto DCAF1, a substrate receptor subunit of the CRL4 E3 ubiquitin ligase, leading to SAMHD1 polyubiquitination and proteasomal degradation (21). This general mechanism appears to be conserved, because divergent Vpx and Vpx-like proteins from a broad range of primate lentivirus lineages possess the ability to deplete SAMHD1 proteins from their cognate primate host species (22, 25).

Loss of function mutations in the SAMHD1 locus are associated with an autoimmune condition termed Aicardi-Goutieres syndrome (AGS) (26, 27). AGS is a rare, inherited, inflammatory disorder whose clinical and immunologic features resemble a congenital viral infection (28, 29). AGS is caused by mutations in *SAMHD1* and four other loci encoding the cellular 3′-5′ exonuclease TREX1 and subunits of the RNaseH2 complex. It is thought that AGS proteins prevent inappropriate triggering of an innate immune response to self-nucleic acids (29–31), either by preventing the synthesis of dead-end products of cellular nucleic acid metabolism, by SAMHD1, or by disposing them, by TREX1 and RNaseH2. To this end, SAMHD1 depletes dNTP pools in terminally differentiated nondividing innate immune cells, thereby ensuring that endogenous, noncanonical, potentially immunostimulatory nucleic acids will not be synthesized in cells possessing active innate nucleic acid sensing and effector systems.

The catalytic activity of SAMHD1, residing in the central HD domain, is essential for its ability to inhibit HIV infection in monocytic cells (13). Previous studies provided evidence that neither the conserved SAM domain nor SAMHD1 nuclear localization is required for SAMHD1 to inhibit HIV infection (19, 20). Little is known, however, about the interrelation(s) between other SAMHD1 domains and additional requirements for effective anti-viral and cellular/innate immune functions. To address these issues, we initiated structure-function and biochemical studies of the human SAMHD1 protein. Interestingly, our studies show that SAMHD1 oligomerizes and forms tetramers, reveal that SAMHD1 tetramerization is modulated by elements flanking the catalytic core domain, and link the tetrameric state to SAMHD1 catalytic and anti-viral activities.

## EXPERIMENTAL PROCEDURES

### 

#### 

##### Mammalian Expression Constructs and Viruses

Human SAMHD1 deletion and point mutants were constructed using standard techniques and subcloned into pCG plasmids (32) encoding N-terminal Myc, HA, or FLAG epitope tags, and/or into MSCV(puro) retroviral vector. Vesicular stomatitis virus glycoprotein pseudotyped MSCV(puro) viral particles were produced from transiently transfected HEK 293T cells (33). SIV virus-like particles loaded with Vpx were produced as described previously (5, 12). A single cycle HIV-1 luciferase reporter construct HIV-1 NL4–3-Luc-R^−^E^−^, constructed by N. Landau (34), was provided by Tom Hope and David McDonald.

##### SAMHD1 Restriction Assays

U937 cells (2 × 10^5^) were transduced with MSCV(puro) retroviral vectors expressing wild type or mutant, epitope-tagged SAMHD1. Three days after infection, the cells were plated in 24-well plates in the presence of 100 ng/ml phorbol 12-myristate 13-acetate (PMA) to initiate their differentiation to macrophages. Two days later, the cells were rested for a day in fresh medium and then challenged with a single cycle HIV-1 NL4–3-Luc-R^−^E^−^ expressing the luciferase reporter, alone or co-infected with SIV virus-like particles loaded with Vpx. Luciferase activity was quantified 48 h later with a luciferase assay system (Promega).

##### LC-MS Quantification of dNTPs

dNTPs were extracted from PMA-differentiated U937 cells (5 × 10^5^) and evaporated under vacuum at 70 °C (17). The dried material was resuspended in 100 μl of H_2_O, mixed with [^13^C^15^N]dNTP standard, and analyzed with the Agilent Technologies 1100 series HPLC system (Agilent Technology, Santa Clara, CA) interfaced with the LXQ linear ion trap mass spectrometer equipped with an electrospray ionization source (Thermo Scientific, Waltham, MA). HPLC separation of dNTPs was achieved on Hypersil GOLD-C18 column 100 × 1 mm, 3-μm particle size (Thermo Electron, Waltham, MA) in a linear gradient (0–60%) of acetonitrile in 20 mm water solution of ammonium bicarbonate containing 3 mm hexylamine, pH 9.2. The gradient was developed over 15 min at a flow rate of 0.2 ml/min (35). The mass spectrometer was operated in the positive ionization mode. To achieve appropriate sensitivity, the instrument parameters were tuned using dATP in the corresponding mobile phase and a flow rate. The ion intensity for dATP was recorded in selected ion monitoring mode using 593.0 [MH + 101 hexylamine]^+^ *m*/*z* value, which corresponds to dATP-hexylamine adduct. The analyte was quantified based on the reference peak of added isotopically labeled internal standard (^13^C_10_^15^N_5_]dATP, *m*/*z* = 607.1 [MH + 101 hexylamine]^+^; Sigma-Aldrich). The correction factor was calculated for the analyte/internal standard ion pair using the ratio of the areas of ion intensities for the analyte and the internal standard area in a concentration range of 2–400 nm.

##### Western Blotting

Cell extracts were separated by SDS-PAGE and transferred to PVDF membrane for immunoblotting. Proteins were detected with monoclonal antibodies specific for HA, FLAG, or Myc epitope tags, and immune complexes were revealed with HRP-conjugated antibodies specific for the Fc fragment of mouse or rabbit immunoglobulin G (1:5,000; Jackson ImmunoResearch Laboratories) and enhanced chemiluminescence (Amersham Biosciences) or revealed with fluorescent antibodies to mouse or rabbit immunoglobulin G (Kirkegaard and Perry Laboratories) and Odyssey Infrared Imager (Licor).

##### Formaldehyde Cross-linking and Immunoprecipitation

U937 cells expressing HA-FLAG-AU1- (hfa-)tagged SAMHD1 were incubated at 37 °C for 10 min in the presence of the indicated concentrations of formaldehyde in culture medium (RPMI 1640 medium, 10% fetal bovine serum). Cross-linking was terminated by the addition of glycine to a final concentration of 0.125 m. Cells were washed with cold PBS and extracted with RIPA buffer (10 mm Tris-HCl, pH 8.0, 1% Triton X-100, 0.1% SDS, 0.5% sodium deoxycholate, 1 mm EDTA, 140 mm NaCl) containing protease inhibitors (Roche Applied Science), and SAMHD1 was immunoaffinity purified by two sequential immunoprecipitations via HA and FLAG epitope tags, each followed by competitive elution with epitope peptide (36). Cross-linking of SAMHD1 complexes purified from U937 cells was partially reversed by incubating the samples at 75 °C for 60 min, in the presence of SDS sample buffer.

##### Escherichia coli SAMHD1 Expression and Purification

The cDNAs encoding wild type and mutant SAMHD1 proteins were cloned into the pET28 vector (EMD4Biosciences) with a His_6_ tag at the N terminus, expressed in *E. coli* Rosetta 2 (DE3) cultured in Luria-Bertani medium using 0.4 mm isopropyl β-d-thiogalactopyranoside for induction at 18 °C for 16 h, and purified as described previously (21). The average variation in SAMHD1 dNTPase specific activity calculated for four independent SAMHD1 batches was ±5%.

##### Analytical Size Exclusion Column Chromatography of Protein Complexes

100 μl of purified proteins or protein mixtures (10 μm) with dGTP at the indicated concentrations in 20 mm Tris-HCl, pH 7.8, containing 50 mm NaCl, 1 mm CaCl_2_, 1 mm DTT, 5% glycerol, and 0.02% sodium azide were injected into a 24-ml analytical Superdex 200 column (1 × 30 cm; GE Healthcare) and separated at a flow rate of 0.8 ml/min in the same buffer without reducing agent. The absorbance at UV280 was recorded, and 0.5 ml peak fractions were collected and assayed for dNTPase activity. Alternatively, 100 μl of proteins at 250 nm in mixture with dGTP at indicated concentrations (0–200 μm) were injected into the analytical Superdex 200 column equilibrated with Tris-HCl, pH 7.8, 50 mm NaCl, 5% glycerol, and 0.02% sodium azide. The elution profiles were recorded by monitoring fluorescence trace with excitation at 282 nm and emission at 313 nm.

##### Multiangle Light Scattering (MALS)

The molecular masses of protein or protein complexes were determined using an analytical Superdex 200 column with in-line multiangle light scattering (HELEOS; Wyatt Technology), variable wavelength UV (Agilent 1100 series; Agilent Technology), and refractive index (Optilab rEX; Wyatt Technology) detector. Typically, 100 μl of protein solution at 2 mg/ml was injected into the column equilibrated with 20 mm Tris-HCl buffer, pH 7.8, containing 50 mm NaCl, 1 mm CaCl_2_, and 0.02% sodium azide at a flow rate of 0.5 ml/min at room temperature. Prior to column chromatography, proteins were incubated with dGTP, as indicated, in the same buffer containing 1 mm DTT. The ASTRA program (version 5.3.4; Wyatt Technology) was used for light scattering data analysis.

##### SAMHD1 dGTP-dependent dNTPase Assay and Formaldehyde Cross-linking

dNTPase assays were carried out in a reaction buffer containing 10 mm Tris-HCl, pH 7.5, 50 mm NaCl, 5 mm MgCl_2_, appropriate dNTPs each at 100 μm, and 56 nm (28 pmol) recombinant SAMHD1 at 25 °C. Aliquots collected at various time points were diluted into 9 volumes of ice-cold PBS to stop the reaction and spun through an Amicon Ultra 0.5-ml 10 kDa filter (Millipore) at 14,000 × *g* for 20 min. Deproteinized samples were analyzed by HPLC using an Eclipse XDB-C18 4.6- × 150-mm column (Agilent). The column was equilibrated in 0.1 m KH_2_PO_4_, 10 mm tetrabutylammonium bromide, pH 6.5 (Solvent A). Injected samples were eluted with a linear gradient of 0–7.5% acetonitrile in solvent A, over 30 min followed by 7.5% acetonitrile in solvent A for 20 min at flow rate of 1 ml/min. Alternatively, deoxyribonucleosides and dNTPs were separated over a CapCell Pak C18 4.6- × 250-mm column (Phenomenex) pre-equilibrated with 10 mm ammonium phosphate, pH 7.8, and 4.8% methanol. Then they were eluted with a linear gradient of 4.8–19.2% methanol over 22.5 min at a flow rate of 1.5 ml/min. The amounts of substrates and products were quantified by peak integration of the absorbance data recorded at 260 nm. For chemical cross-linking experiments, the reactions were assembled in the absence or presence of the indicated dGTP concentrations and preincubated on ice for 30 min, diluted with equal volume of 2% formaldehyde solution in PBS to a final concentration of 1%, followed by incubation for 15 min at room temperature. The cross-linking reactions were quenched with 0.25 m glycine for 15 min at room temperature, resolved by SDS-PAGE, and SAMHD1 was revealed by silver staining.

## RESULTS

### 

#### 

##### SAMHD1 Regions Located Outside of the Catalytic HD Domain Modulate Inhibition of HIV-1 Infection

**FIGURE 1.**
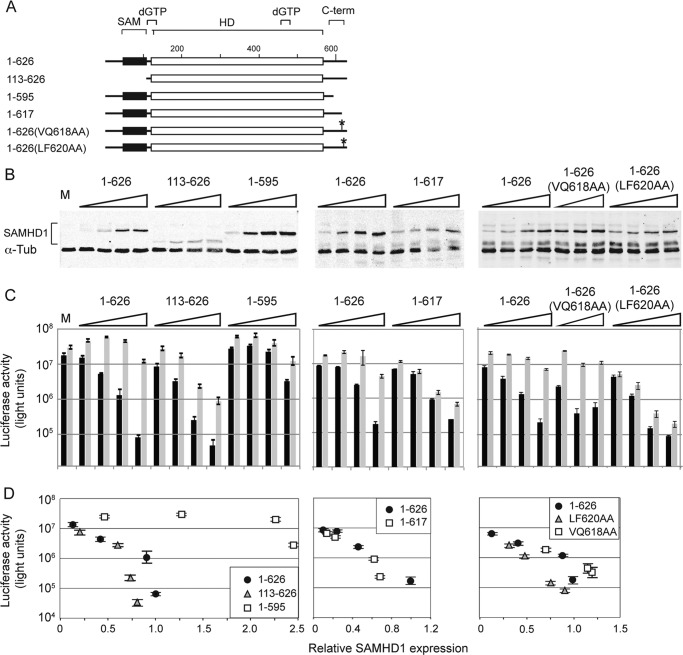
**SAMHD1 mutations located outside the catalytic domain modulate its ability to inhibit HIV-1 infection.** *A*, schematic representation of the human SAMHD1 protein (626 residues). The locations of the SAM domain, allosteric dGTP effector binding site (dGTP), the HD/COG1078 phosphohydrolase domain (HD), and the divergent C terminus (*C-term*), which is targeted by SIVmac/HIV-2 Vpx, are shown. *B*, expression of SAMHD1 protein variants in U937 cells. Aliquots of cell extracts used for the luciferase assays shown in *C* were immunoblotted for SAMHD1 via its N-terminal epitope tag, and α-tubulin (α*-Tub*), as a loading control. U937 cells transduced with an empty MSCV puro vector (*M*) were used as a negative control. Fluorescent signals were quantified with an Odyssey infrared imaging system. *C*, effect of SAMHD1 mutations on its ability to inhibit HIV-1 infection. Wild type and mutant SAMHD1 proteins were expressed in U937 cells by retroviral transduction over a range of multiplicities of infection in dose-response experiments. The transduced cells were induced to differentiate toward macrophages and then challenged with a single cycle vesicular stomatitis virus glycoprotein pseudotyped HIV-1 carrying a luciferase reporter gene, in the absence (*black bars*) or presence (*gray bars*) of SIV virus-like particles loaded with Vpx (4, 12) in triplicate wells. Luciferase activity in cell extracts was quantified 2 days later and is shown in arbitrary units, with ± 1 σ error bars. *D*, mutations outside the HD domain modulate SAMHD1 potency. The scatter plots show luciferase reporter activity (from *C*) as a function of SAMHD1 variant protein expression level (*B*) normalized to that of the wild type protein.

SAMHD1 possesses a conserved N-terminal SAM domain, an HD dNTP triphosphohydrolase domain, which comprises a binding site for an allosteric activator, dGTP as well as the catalytic site, and a C-terminal region that is highly divergent in SAMHD1 proteins from different vertebrate species (Fig. 1*A*). Whereas a functional HD domain is essential for the ability of SAMHD1 to inhibit primate lentivirus infection in nondividing cells (13), the roles of other SAMHD1 domains are not well established. To address their importance, we constructed a set of human SAMHD1 deletion mutants, shown in Fig. 1*A*, and quantified their ability to inhibit HIV-1 infection in U937 monocytes.

Wild type and mutant SAMHD1 proteins, tagged on their N termini with tandem HA, FLAG, and AU1 epitopes (hfa epitope tag), were expressed in U937 cells by retroviral transduction. Infections were carried out over a range of multiplicities of infection, to study SAMHD1 restriction over a broad range of SAMHD1 expression levels (Fig. 1*B*). Three days following SAMHD1 transduction, U937 cells were exposed to PMA to induce their differentiation toward macrophages and then challenged with a single cycle vesicular stomatitis virus glycoprotein pseudotyped HIV-1 reporter virus harboring a luciferase reporter gene in the *nef* locus (34). As shown in Fig. 1*C* (*left panel*), wild type SAMHD1(1–626) potently inhibited HIV-1 infection, as revealed by a more than 100-fold reduction in the luciferase reporter activity in U937 cells expressing SAMHD1 at the highest level, compared with the luciferase activity in control, untransduced U937 cells. A scatter plot of luciferase activity as a function of SAMHD1 level (Fig. 1*D*) clearly illustrates the dose-dependent nature of the inhibition. To confirm that the inefficient infection of the cells reflected SAMHD1-mediated inhibition, the cells were exposed to SIV virus-like particles loaded with Vpx, which programs SAMHD1 for proteasomal degradation (12–14). This treatment relieved, to a large extent, the inhibitory effect (Fig. 1*C*), thus corroborating that it was indeed mediated by SAMHD1.

Several sequences located outside the catalytic domain appeared to positively or negatively modulate the ability of SAMHD1 to restrict HIV-1 infection (Fig. 1*D*). The most striking effect was observed with a SAMHD1 C-terminal deletion variant, SAMHD1(1–595), which was unable to efficiently inhibit HIV-1 infection (Fig. 1*D*), even when expressed at levels similar to those at which full-length SAMHD1 suppressed HIV-1 infection by more than 100-fold. In contrast, a variant with a smaller C-terminal deletion, SAMHD1(1–617), was able to efficiently restrict HIV-1 infection. Of note, both C-terminal deletions disrupt the Vpx docking site on SAMHD1 (21) and thereby render these mutant proteins resistant to depletion by Vpx. This explains the apparent lack of Vpx-mediated relief of the inhibitory effect of the SAMHD1(1–617) variant. Interestingly, deletion of the N terminus, removing the conserved SAM domain in SAMHD1(113–626), resulted in a slight, yet reproducible enhancement of the ability of SAMHD1 to inhibit HIV-1 infection, which was relieved by Vpx, consistent with previous reports that this mutant is targeted for degradation by HIV-2/SIVmac Vpx, albeit less efficiently than full-length SAMHD1 (19–21, 37). A similar enhancement of the anti-HIV activity of SAMHD1 was displayed following double alanine substitution of a well conserved hydrophobic motif (leucine 620 and phenylalanine 621) that is present in the divergent C termini of vertebrate SAMHD1 proteins (see supplemental Fig. 1*C* in Ref. 22). This variant, SAMHD1(LF620AA), exhibited a consistent, slightly enhanced inhibition of HIV-1, whereas alanine substitutions for the preceding nonconserved amino acid residues, valine 618 and glutamine 619, in SAMHD1(VQ618AA), did not have such an effect. These latter point mutations were derived from an alanine scan screen of the SAMHD1 C terminus for mutations modulating inhibition of HIV-1 infection (data not shown). Together, the above observations revealed that SAMHD1 sequences located outside the catalytic domain and not known to be directly involved in dNTP hydrolysis modulate the ability of SAMHD1 to inhibit HIV-1 infection and raised the question as to how they exert their controlling effects.

##### SAMHD1 C Terminus Is Not Important for dNTP Phosphohydrolase Activity in Vitro

SAMHD1 is thought to inhibit HIV-1 infection by depleting dNTP pools in nondividing cells to levels too low to be conducive for synthesis of proviral cDNA by viral reverse transcriptase (23, 24). Therefore, the requirement for SAMHD1 C-terminal sequences in HIV-1 inhibition and the enhancement of inhibition by other sequences (Fig. 1) prompted us to explore whether any of these, in particular the SAMHD1 C terminus, has a role in modulating dNTPase activity.

**FIGURE 2.**
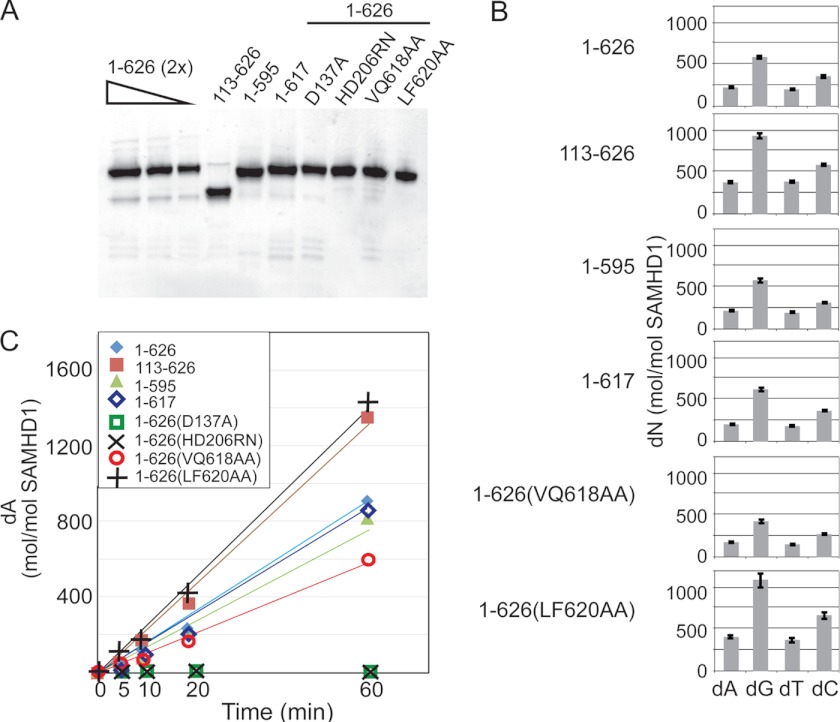
***In vitro* dNTPase activity of recombinant SAMHD1 proteins.** *A*, 150-ng aliquots of SAMHD1 protein variants were separated on Novex 4–12% Bis-Tris gradient gels along with serial 2-fold dilutions of full-length SAMHD1 standards (1–626 (2x); 300, 150, and 75 ng) and revealed by silver staining. *B*, mutations do not alter SAMHD1 preference for dNTP substrates. Reactions containing wild type (1–626) or the indicated mutant SAMHD1 proteins and 100 μm each dATP, dGTP, dCTP, and TTP were incubated in the presence of 5 mm MgCl_2_ for 20 min and deoxyribonucleoside (*dN*) products of the reactions quantified by HPLC. The average amounts of dA, dG, dT, and dC generated in each reaction, from triplicate samples in two experiments ± standard error, expressed as deoxyribonucleoside/SAMHD1 (mol/mol), are shown. *C*, dGTP-dependent dATPase activity of wild type and mutant SAMHD1 proteins. SAMHD1 proteins were incubated with dGTP, dATP, dCTP, and TTP for the indicated times, and the amount of dA generated at each indicated time is shown. The standard error from triplicate samples was <5% and usually <2% of the values shown.

We began by investigating the effect of selected mutations of SAMHD1 on its dNTP triphosphohydrolase activity *in vitro*, using recombinant SAMHD1 proteins expressed in *E. coli*. Wild type and mutant SAMHD1 proteins, purified to near homogeneity (Fig. 2*A*), were incubated with a mixture of dATP, dGTP, dCTP, and TTP, and then deoxyribonucleoside products of the reactions were quantified by HPLC. As shown in Fig. 2*B*, the full-length SAMHD1 protein showed distinct preferences for individual dNTP substrates with dGTP > dCTP > dATP/TTP, consistent with previous reports (17, 18). Significantly, the dNTPase activity of the SAMHD1(1–595) mutant was similar to that seen for the full-length SAMHD1(1–626), indicating that the C terminus is not required for SAMHD1 catalysis under the conditions of this assay (Fig. 2*C*). Interestingly, the LF620AA substitution in the C terminus resulted in an increased rate of dNTP hydrolysis by as much as 50%, as did a deletion of the N-terminal region including the SAM domain (Fig. 2*C*), suggesting that these latter elements can modulate SAMHD1 catalytic activity, consistent with our finding that these same mutations enhance HIV-1 infection inhibition by HIV-1. Further, SAMHD1 proteins harboring amino acid substitutions at the predicted allosteric dGTP effector binding site (D137A) or catalytic center (HD206DN) (17) did not show any catalytic activity, thus validating the assay. Despite some experimental variability (up to 20–30%), these findings were corroborated through several independently performed assays (data not shown). Of note, none of the mutations detectably affected the order of substrate preference (Fig. 2*B*), consistent with their location away from the substrate binding/active site.

##### SAMHD1 C Terminus Is Important for Full Capacity to Deplete dNTPs Levels in Vivo

**FIGURE 3.**
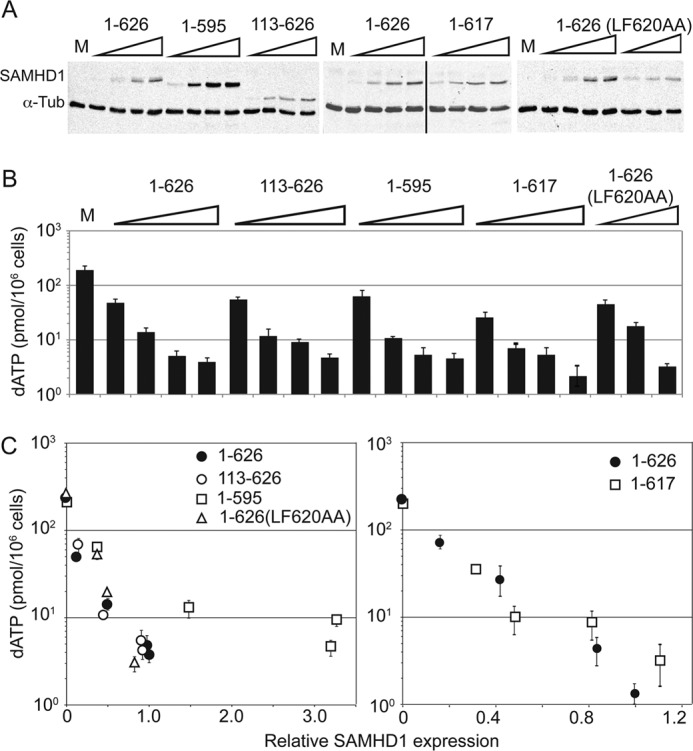
**SAMHD1 C terminus is required for efficient depletion of dATP *in vivo*.** *A*, *in vivo* activity of mutant SAMHD1 proteins. U937 cells expressing SAMHD1 wild type or variants over a range of levels were induced to differentiate toward macrophages, and dATP pools were determined by LC-MS 3 days later. The average dATP level/10^6^ cells from triplicate samples in three representative experiments ± standard error is shown for each SAMHD1 variant. α*-Tub*, α-tubulin. The *vertical line* in the *middle panel* indicates the place where the two parts of this blot are joined. *B*, SAMHD1 levels in U937 cells. Aliquots of extracts from SAMHD1-expressing U937 cells used for the dATP determinations shown in *A* were immunoblotted for SAMHD1, via its N-terminal epitope tag, and α-tubulin, as a loading control. Fluorescent signals were quantified using an Odyssey infrared imaging system. *M*, control U937 cells transduced with empty MSCV vector. *C*, *in vivo* potency of mutant SAMHD1 proteins. For each SAMHD1 variant, the amount of dATP, expressed as pmol/10^6^ cells (from *A*), is plotted as a function of SAMHD1 expression level (from *B*), normalized to that of wild type SAMHD1.

Next, we asked whether the SAMHD1 C terminus is required for effective depletion of dNTPs *in vivo* in differentiated U937 cells. The experimental approach similar to that described above for assaying SAMHD1 restriction capacity was used (Fig. 1). Specifically, wild type and mutant SAMHD1 proteins were expressed to different levels in U937 cells by retroviral transduction at appropriate multiplicities of infection and then induced to differentiate by exposure to PMA. Three days later, cells were harvested to quantify dNTP pools by coupled LC-MS and SAMHD1 levels by Western blotting. The LC-MS assay permitted detection of dATP pools but was not sensitive enough to allow accurate quantification of dGTP, dCTP, and TTP levels in SAMHD1-expressing differentiated U937 macrophages (data not shown). Because the intrinsic SAMHD1 preference toward dNTP substrates was unaltered by any tested mutation (Fig. 2*B*), we used dATP levels as the readout for SAMHD1 activity *in vivo*. Expression of full-length SAMHD1 protein as well as each of the variant SAMHD1 proteins resulted in a dose-dependent decrease of dATP levels, by up to ∼100-fold (Fig. 3). Scatter plots presenting cellular dATP pool size as a function of SAMHD1 expression level, shown in *panel C*, revealed that most of the analyzed mutations did not detectably impair the ability of SAMHD1 to deplete cellular dATP, consistent with our previous finding that they retained the ability to fully inhibit HIV-1 infection. One notable exception was the SAMHD1(1–595) variant, whose ability to deplete dATP levels in U937 cells appeared to be reduced. In particular, much higher levels of this variant, compared with those of wild type and other mutant SAMHD1s tested, were required to deplete dATP levels to the same extents. This finding revealed that the C terminus promotes depletion of dNTP levels by SAMHD1 *in vivo* and provided a tentative explanation as to why this SAMHD1 variant was ineffective in protecting cells from HIV-1 infection (Fig. 1).

##### dGTP Binding at the Predicted Allosteric Site Induces SAMHD1 Tetramerization

**FIGURE 4.**
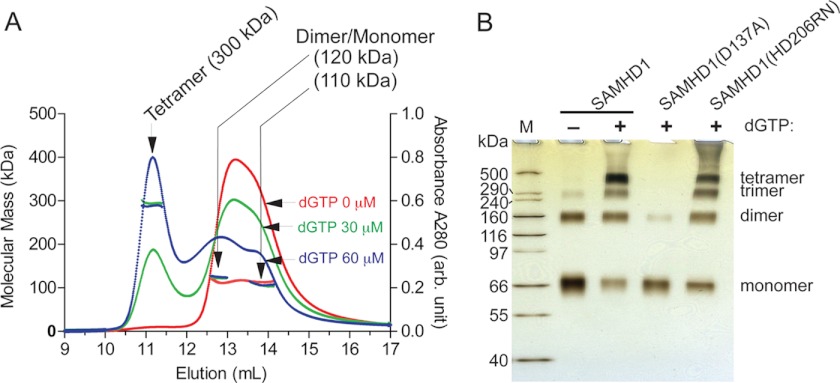
**dGTP induces SAMHD1 tetramerization.** *A*, characterization of quaternary states of recombinant SAMHD1 by SEC-MALS. SAMHD1 (25 μm) alone (*red trace*) was injected into an analytical gel filtration column at a flow rate of 0.5 ml/min, and the absorbance trace was recorded at 280 nm. The absorbance (*right y axis*) and MALS molecular mass profile (*left y axis*) across the elution volume are shown. The average molecular mass of species eluting between 12.6 and 14.2 ml was estimated to be 110 kDa, by MALS. Of note, the calculated molecular mass of SAMHD1 is 75 kDa. Mixtures of SAMHD1 with two different concentrations of dGTP (30 μm, *green trace*; and 60 μm, *blue trace*) were also analyzed by SEC-MALS. The elution volumes for tetrameric SAMHD1 species (300 kDa) and a mixture of dimeric and monomeric protein species (110–120 kDa), estimated by MALS, are indicated by *arrows. B*, SDS-PAGE profiles of chemically cross-linked dGTP-induced SAMHD1 oligomers. Recombinant wild type and variant SAMHD1 proteins with mutations in the predicted dGTP-binding allosteric site (D137A) or catalytic center (HD206RN) (17) were preincubated with dGTP in a reaction buffer, as indicated, chemically cross-linked, and separated on 4–12% Novex gels, and the proteins were visualized by silver staining. The positions of protein bands corresponding to SAMHD1 oligomers and molecular masses of proteins in HiMark protein standards (*M*) are indicated.

Previous structural and biochemical studies of human SAMHD1 fragment (residues 120–626) suggested that the catalytically active form of full-length SAMHD1 is a dimer allosterically activated by dGTP binding (17). The role of SAMHD1 C terminus has been unknown, because this region was not resolved in the crystal structure. We speculated that the C terminus mutation could affect some possibly subtle aspect of SAMHD1(1–595) self-interaction and thereby interfere with its function *in vivo*. To address this possibility, we first assessed the quaternary state of recombinant full-length SAMHD1 in the presence or absence of dGTP, using SEC-MALS. In the absence of dGTP, SAMHD1 eluted in a single peak (Fig. 4*A*, *red trace*). The average molecular mass of the protein eluting in the main peak was estimated to be ∼110 kDa by MALS. Because the mass of recombinant SAMHD1 monomer containing an N-terminal His_6_ tag and a short linker was calculated to be 75 kDa, and it was reported that SAMHD1 (residues 120–626) crystallizes as a dimer in the absence of dGTP (17), this peak probably represented an unresolved mixture of SAMHD1 monomers (75 kDa) and dimers (150 kDa). Strikingly, preincubation with 30 μm dGTP resulted in a more complex elution profile. Whereas the majority of SAMHD1 (∼70–80%) eluted in volumes expected of SAMHD1 monomers and dimers, the remaining protein eluted significantly earlier (11.1 ml; Fig. 4*A*, *green trace*). Preincubation in the presence of 60 μm dGTP (Fig. 4*A*, *blue trace*) resulted in a similar elution profile except that a larger fraction of SAMHD1 eluted early in the dGTP-induced peak. Importantly, preincubation with 4 mm dGTP shifted essentially all SAMHD1 into the early eluting peak, thus indicating that the vast majority of the protein was capable of forming these large dGTP-induced complexes (supplemental Fig. S1). The molecular mass of protein in this peak was estimated to be 300 kDa by MALS, suggesting that dGTP induces SAMHD1 tetramerization.

**FIGURE 5.**
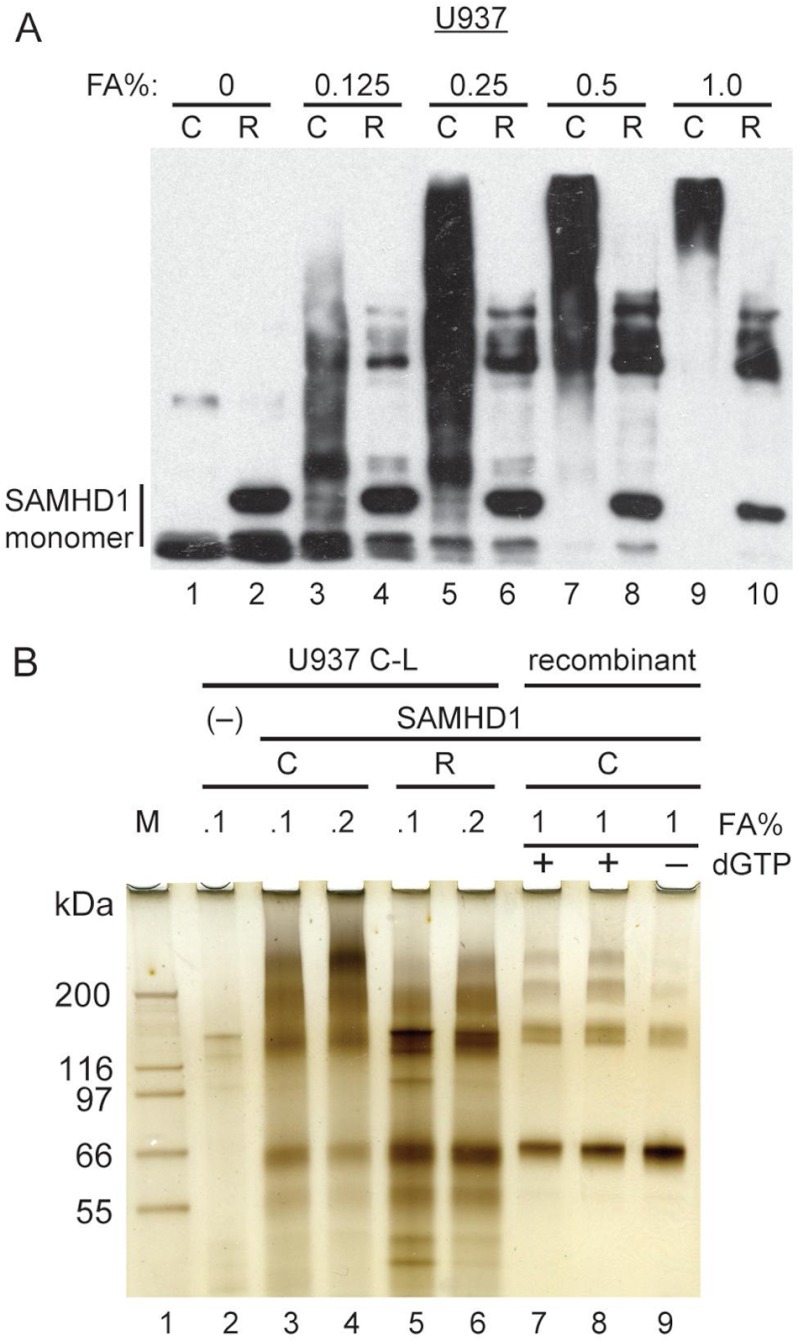
**SAMHD1 oligomers in intact cells.** *A*, SAMHD1 is oligomeric in intact U937 cells. U937 cells expressing epitope-tagged hfa-SAMHD1 were cross-linked (*C*) with formaldehyde at the indicate concentrations (*FA*%). Aliquots of cell extracts supplemented with gel loading buffer were separated by SDS-PAGE and blotted with FLAG-M2 antibody reacting with the FLAG tag at the N terminus of SAMHD1. A subset of samples was heated at 90 °C for 20 min to partially reverse the cross-links (*R*) prior to separation and blotting. Bands corresponding to monomeric and slower migrating cross-linked forms of SAMHD1 are indicated. *B*, characterization of cross-linked SAMHD1 forms purified from U937 cells. hfa-tagged SAMHD1 was purified from U937 cells cross-linked with 0.1 or 0.2% formaldehyde, by two sequential immunoprecipitations via HA and FLAG tags. Cross-links were partially reversed (*R*; *lanes 5* and *6*) or not (*C*; *lanes 2–4*), and the proteins were resolved as described for *A* above. A mock immunoprecipitate from U937 cells not expressing SAMHD1 was used as a negative control (*lane 2*). Recombinant SAMHD1 protein cross-linked with formaldehyde in the presence (*lanes 7* and *8*) or absence (*lane 9*) of dGTP was resolved in parallel, as control. Molecular masses of proteins in Mark12 protein standards (M) are indicated. Protein bands were revealed by silver staining.

To corroborate the findings from SEC-MALS analyses, formaldehyde cross-linking experiments were performed, and the cross-linked proteins were analyzed by SDS-PAGE. As shown in Fig. 4*B*, recombinant SAMHD1 that was cross-linked in the absence of dGTP migrated as a monomer, with a small amount in a slower migrating band that could correspond to SAMHD1 dimer. In contrast, cross-linking performed in the presence of dGTP resulted in a ladder of oligomeric forms up to a tetramer, but not higher oligomers, with apparent migration rates roughly consistent with the SEC-MALS data. Of note, the SAMHD1 tetramer content was likely underestimated because of inefficient cross-linking, because increasing formaldehyde concentration resulted in a higher yield of SAMHD1 oligomers (data not shown; see also Fig. 5*A*). To confirm that SAMHD1 tetramerization indeed required dGTP binding to the allosteric site, similar analyses were performed with SAMHD1 proteins harboring amino acid substitutions at the predicted allosteric dGTP effector binding site (D137A) or, as a control, catalytic center (HD206DN) (17). The disruption of the allosteric dGTP-binding site largely disrupted dGTP-induced SAMHD1 oligomerization, whereas mutation at the catalytic site did not have such an effect (Fig. 4*B*). We conclude that full-length SAMHD1 oligomerizes in a dGTP-dependent manner up to a tetramer *in vitro*.

##### SAMHD1 Is Oligomeric in Vivo in Intact Cells

We next tested whether SAMHD1 tetramers/oligomers can be detected in a more physiological setting by performing chemical cross-linking in intact cells. U937 cells expressing hfa-tagged SAMHD1 protein were cross-linked with formaldehyde at increasing concentrations. Cell lysates prepared from the cross-linked cells were either heated at 90 °C, to partially reverse the cross-links, or left untreated, resolved by SDS-PAGE, and immunoblotted for SAMHD1 with anti-FLAG antibody. The cross-linking resulted in slower migration of FLAG antibody reactive material (Fig. 5*A*), suggestive of SAMHD1 participation in high molecular weight protein complexes. Strikingly, partial reversal of the cross-links revealed a set of discrete high molecular weight bands reactive with anti-FLAG antibody, reminiscent of dGTP-induced oligomers seen upon cross-linking of recombinant SAMHD1.

To corroborate that the slowly migrating SAMHD1 forms detected in cross-linked cells were indeed SAMHD1 oligomers, SAMHD1 complexes were purified from formaldehyde cross-linked U937 cells by two sequential immunoprecipitations via FLAG and HA epitope tags (36) and separated by SDS-PAGE side by side with cross-linked recombinant SAMHD1 oligomers, and proteins were visualized by silver staining. It can be clearly seen in Fig. 5*B* that both the U937 and recombinant *E. coli* purified SAMHD1 complexes displayed similar electrophoretic migration patterns. Small differences between these patterns probably reflect different cross-link densities, because a partial cross-link reversal resulted in slightly faster migration of the of U937 cell-derived SAMHD1 complexes (Fig. 5*B*, compare *lanes 3* and *4* with *lanes 5* and *6*). We conclude that SAMHD1 oligomers, including tetramers, are the dominant forms of SAMHD1 *in vivo*.

##### SAMHD1 Tetramer Is the Catalytically Active dNTP Phosphohydrolase

**FIGURE 6.**
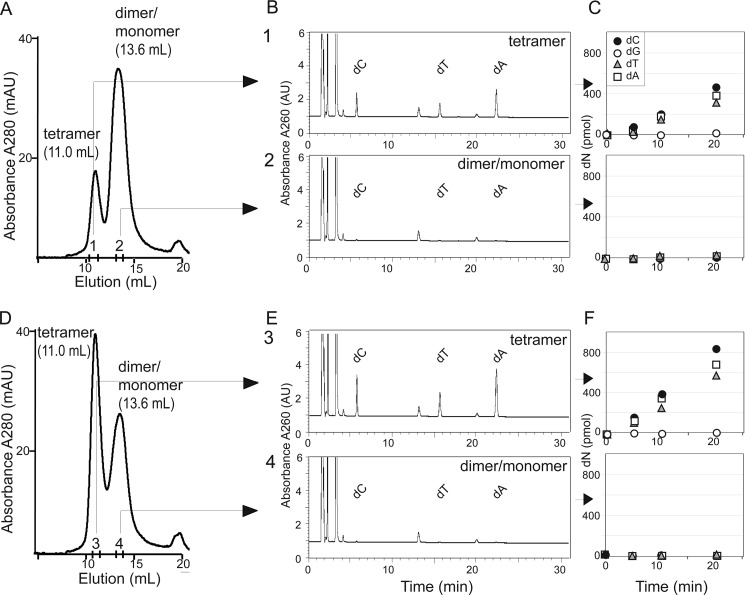
**Tetrameric SAMHD1 is catalytically active dNTPase.** *A* and *D*, recombinant SAMHD1 (10 μm) was incubated with 30 μm (*A*) or 60 μm (*D*) dGTP and separated by size exclusion chromatography at a flow rate of 0.8 ml/min. Elution profiles (*A*_280 nm_) with positions of SAMHD1 tetramer and dimer/monomer peaks indicated are shown. Pooled fractions corresponding to the tetramer (*positions 1* and *3*; elution volumes 10.5–11.0 ml) and dimer/monomer mixture (*positions 2* and *4*; elution volumes 13.25–13.75 ml) peaks were pooled and assayed for dNTPase activity, shown in *B* and *E* and in *C* and *F*, respectively. *B*, *C*, *E*, and *F*, equal volume aliquots (300 μl) of pooled SAMHD1 tetramer or monomer peak fractions were incubated with 100 μm each dATP, dCTP, and TTP in the reaction buffer for the indicated times, and deoxyribonucleoside (*dN*) products of the reactions quantified by HPLC (*C* and *F*). Of note, dGTP was not added to dNTPase assay mixtures. Chromatograms showing separation of the reaction products, collected at the 20-min time point, are shown in *B* and *E*. The results of experiments performed with SAMHD1 oligomers formed in the presence of 30 μm dGTP and 60 μm dGTP are shown in *B* and *C* and in *E* and *F*, respectively.

The D137A mutation predicted to disrupt dGTP binding at the allosteric site (17) abolished dGTP-induced activation of SAMHD1 catalytic activity and tetramerization. Interestingly, EF1143, a bacterial HD domain dNTPase, is a tetramer (38). Hence, we reasoned that full-length SAMHD1 tetramer is likely to be the catalytically active form of this dNTPase. To scrutinize this, recombinant SAMHD1 was preincubated with dGTP (30 μm) to induce tetramer formation as described above. Next, the reaction was fractionated by size exclusion column chromatography, and the dNTPase activity in pooled SAMHD1 fractions, corresponding to tetramer or dimer/monomer, was assayed on a mixture of dATP, dCTP, and TTP (Fig. 6, *A–C*). The range of tetramer and dimer/monomer fractions were defined based on SEC-MALS data (Fig. 4*A*). The MALS data suggested that a 10.5–11.0-ml fraction should contain SAMHD1 tetramer and be free of dimer/monomer, whereas the 13.25–13.75-ml fraction mainly comprises dimer and monomer (Fig. 4*A*). dNTP hydrolysis was only observed in fractions containing SAMHD1 tetramer (Fig. 6, *B* and *C*). Strikingly, the rate of hydrolysis appeared constant through at least the first 20 min of the reaction (Fig. 6C). Because the only source of the allosteric dGTP activator in the reactions was that bound to SAMHD1, this observation suggests that the dGTP dissociation rate from the allosteric site is slow or that the nucleotide exchanges among the allosteric sites within the tetramer. Importantly, increasing the dGTP concentration from 30 to 60 μm in the SAMHD1 oligomerization reaction led to a much larger tetramer-containing peak and greater hydrolysis seen with SAMHD1 tetramer, but not dimer/monomer peak (Fig. 6, *D* and *E*). Control experiments demonstrated SAMHD1 in dimer/monomer peak is competent to form tetramer and act enzymatically upon dGTP addition, thus excluding the possibility that dimer/monomer forms lacked catalytic activity caused by misfolding (supplemental Figs. S1 and S2). Furthermore, isolated tetramers converted back to dimer/monomer forms in the absence of dGTP. These results together indicated that the tetramer is the catalytically active form of the SAMHD1 dNTP phosphohydrolase.

##### SAMHD1 C Terminus Stabilizes the Tetramer

**FIGURE 7.**
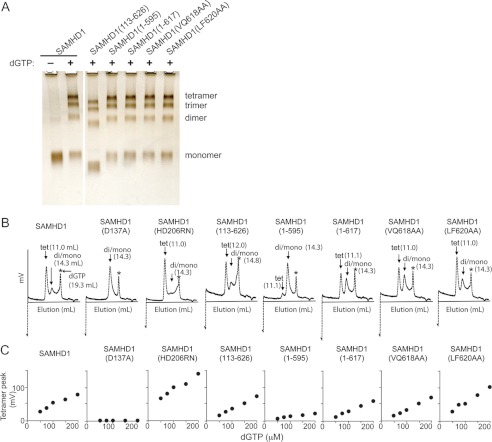
**SAMHD1 C terminus is important for SAMHD1 tetrameric state.** *A*, wild type and the indicated mutant recombinant SAMHD1 proteins were incubated with or without dGTP, chemically cross-linked, separated by SDS-PAGE on 4–12% Novex gels, and visualized by silver staining. *B*, wild type and mutant SAMHD1 proteins (250 nm) were preincubated with dGTP (200 μm), and the mixtures were separated by size exclusion chromatography, as described under “Experimental Procedures.” The elution profiles were recorded by monitoring fluorescence trace (mV, excitation at 282 nm and emission at 313 nm). Peak elution volumes for SAMHD1 tetramer (*tet*) and dimer/monomer mixture (*di/mono*) are indicated (ml), as is the peak corresponding to dGTP, indicated by *asterisks. C*, SAMHD1 regions flanking the catalytic HD domain influence dGTP-induced tetramer. Wild type and mutant recombinant SAMHD1 proteins (250 nm) were preincubated with dGTP at various concentrations (50–200 μm), and the mixtures were injected into an analytical gel filtration column at a flow rate of 0.8 ml/min. The elution profiles (fluorescence trace, excitation at 282 nm and emission at 313 nm) are shown with peak positions corresponding to tetramer and monomer of SAMHD1 indicated. The tetramer peak height (*ordinate*) is plotted as a function of dGTP concentration (*abscissa*).

The SAMHD1(1–595) variant, which lacks the divergent C terminus, was relatively inefficient in depleting the dATP pool *in vivo* (Fig. 3) and a poor inhibitor of HIV-1 infection in U937 cells (Fig. 1). To assess whether this reflected a role for the C terminus in dGTP-induced SAMHD1 tetramerization/oligomerization, chemical cross-linking and size exclusion chromatography experiments were performed. C terminus deletion did not detectably impair the intrinsic ability of SAMHD1(1–595) to form a tetramer, as reported by formaldehyde cross-linking of recombinant protein (Fig. 7*A*). Nor did the other mutations under study, with a possible exception of SAMHD1(113–626), which lacked the N-terminal SAM domain and displayed reduced tetramerization, based on relative band intensity compared with full-length SAMHD1 and other point and deletion mutants.

For a more quantitative assessment of SAMHD1 tetramerization, size exclusion chromatography experiments were performed. SAMHD1 oligomers were allowed to assemble in the presence of dGTP, and the extent of SAMHD1 oligomerization was assessed by SEC. Of note, chromatography was performed without dGTP in the column buffer; hence, this assay was measuring both the initial tetramer assembly as well as its stability during chromatographic separation. This was in contrast to the chemical cross-linking assay, in which dGTP was continuously present during the cross-linking reaction, which possibly moderated tetramer dissociation. As shown in Fig. 7*B*, efficient tetramerization was observed for almost all SAMHD1 variants tested, based on peak elution volumes, in good agreement with the results of chemical cross-linking experiments shown in Fig. 7*A*. One notable exception was the SAMHD1(1–595) mutant, which appeared to be mostly monomeric/dimeric under the conditions of this assay. To further scrutinize and confirm these observations, SAMHD1 oligomers were allowed to assemble in the presence of increasing initial dGTP concentrations, and the mixtures were analyzed by SEC. These dose-response studies, shown in Fig. 7*C*, more clearly revealed that several mutations modulated the propensity of SAMHD1 to tetramerize. Importantly, the SAMHD1(D137A), the predicted allosteric site dGTP-binding mutant failed to form a stable tetramer at any dGTP concentration, consistent with the cross-linking results. Most notably, the yield of tetramer with the SAMHD1(1–595) variant was consistently exceptionally low for all dGTP concentrations tested, compared with other SAMHD1 proteins. This was unlikely to be caused by depletion of dGTP by wild type SAMHD1, because less than 2% of dGTP was converted to dG during tetramer assembly (data not shown). Interestingly, tetramer yield seen with SAMHD1(113–626) and SAMHD1(LF620AA) variants appeared to be slightly, yet consistently, higher than that seen with other SAMHD1 proteins tested. Furthermore, catalytically inactive SAMHD1(HD206RN) also tetramerized to levels higher than wild type SAMHD1. We conclude that both the C terminus as well as other SAMHD1 elements that flank the catalytic domain can modulate the assembly/stability of SAMHD1 tetramers.

## DISCUSSION

SAMHD1, a nuclear dNTPase expressed in major leukocyte populations (12–17), has a dual role. It acts as a potent inhibitor of primate lentivirus infection in terminally differentiated leukocytes and protects the innate immune system from launching an immune response to, presumably autologous, immunostimulatory nucleic acids (12–14, 26, 27). Both actions require functional SAMHD1 dNTPase. This is underscored by previous findings that many AGS-associated mutations disrupt SAMHD1 catalytic activity and that inhibition of HIV infection is abolished by mutating the key histidine and aspartic acid residues in the HD domain catalytic center (13, 26). Conservation of the SAM domain and the ubiquitous presence of a variable C-terminal domain among vertebrate SAMHD1 proteins indicate that they are important for its cellular function. Their precise roles for the anti-viral effects of SAMHD1, however, have not been well defined. To gain new insights into molecular determinants of SAMHD1 function, we assessed its quaternary states both *in vitro* and *in vivo*. We provide evidence implying that SAMHD1 dNTP triphosphohydrolase assembles into tetramers that possess catalytic activity and identify sequence elements located outside of the HD catalytic domain that modulate its function. Our findings reveal the importance of SAMHD1 tetramerization for its biological activity.

It is also evident from our quantitative side by side comparisons of variant proteins that the ability of SAMHD1 to inhibit HIV-1 infection is not dependent on its intracellular localization, confirming and extending previous observations (19, 20). Whereas deleting the N-terminal region of the molecule comprising the nuclear localization signal and the SAM domain causes SAMHD1 to mislocalize to the cytoplasm (19, 20), this does not detectably impair its dNTPase or anti-viral activity. The lack of dependence of SAMHD1 inhibitory effect on its intracellular localization probably reflects high rates of dNTP diffusion from the sites of their synthesis to those of lentivirus replication. The previous finding that the rate of HIV-1 cDNA synthesis is well correlated with the overall dNTP levels in infected cells is also consistent with this possibility (23, 24). Together, this evidence supports the model in which SAMHD1 inhibits HIV-1 infection indirectly, by depleting dNTP pools to levels below the threshold required for viral reverse transcriptase to function efficiently.

Our biochemical and cell-based analyses reveal the oligomeric state of SAMHD1. In the absence of dGTP, we find SAMHD1 to be mostly monomeric and dimeric, and both forms lack detectable catalytic activity. These findings are consistent with previous biochemical characterizations and a structural study that reported the SAMHD1 catalytic core crystallizes as a dimeric unit (17, 18). It is also evident from our physicochemical and cross-linking studies that in the presence of dGTP, SAMHD1 forms tetramers, which are active enzymes. Of note, SAMHD1 trimers observed in cross-linking experiments are likely derived from partially cross-linked tetramers and, as such, lack physiological relevance.

Whereas SAMHD1 tetramers were not detected by analytical ultracentrifugation in a previous report using two catalytically active SAMHD1 fragments (positions 120–626 and 26–583) (17), our findings are not inconsistent with these previous observations. The SAMHD1(23–583) is missing the C terminus and thus is similar to the SAMHD1(1–595) that we found was unable to form stable tetramers in the presence of dGTP (Fig. 7). One possibility is that the SAMHD1(23–585) fragment can form tetramers, but they are unstable, similar to the SAMHD1(1–595) variant characterized in this study. This would explain how the SAMHD1(23–585) fragment can be catalytically active and yet appear to be predominantly dimeric in lengthy analytical ultracentrifugation assays, because of tetramer dissociation upon dGTP depletion. The quaternary states of SAMHD1(120–626) in the absence of dGTP were determined to be monomer and dimer, in equilibrium, again consistent with our observation that tetramer formation is only induced by dGTP binding.

The above findings are corroborated by results of our SAMHD1 cross-linking studies in intact cells, shown in Fig. 5. They clearly document the existence of discrete, higher order SAMHD1 complexes that can be purified and are observed to comigrate with recombinant SAMHD1 oligomers in SDS-PAGE. Notably, such complexes were also detected at similar levels in both actively proliferating and PMA-differentiated U937 cells, *i.e.*, under both restrictive and nonrestricting conditions (data not shown). Together, our findings indicate that SAMHD1 tetramer is the catalytically active form of the enzyme and that this form is prevalent *in vivo* in cell types in which SAMHD1 imposes a barrier to HIV infection.

Available evidence suggests the following model for the functional organization of the SAMHD1 molecule. The centrally positioned HD domain mediates dGTP-dependent SAMHD1 tetramerization, consistent with the results from previous structural analyses of the SAMHD1 catalytic core (17). The distally located regions of the protein modulate SAMHD1 function. In particular, the N-terminal region comprising the nuclear localization signal and a SAM domain likely serves to localize the catalytic domain to specific cellular compartments and processes. Its conservation indicates that compartmentalization is important for the execution of cellular SAMHD1 function(s), even though it is dispensable for its anti-viral effect. The C terminus, distal to the catalytic domain, affects SAMHD1 function by modulating tetramerization. Although the underlying molecular mechanism is unclear at the present time, our data show that the C terminus is important for the maintenance of SAMHD1 tetrameric state *in vitro* and for full capacity to deplete dNTP levels *in vivo*.

The other key role of the SAMHD1 C terminus is to mediate interactions with viral regulators. This region contains a docking site for the Vpx accessory virulence factor of HIV-2 and related strains of primate lentiviruses (21). Vpx binds to this site to load SAMHD1 onto CRL4^DCAF1^ E3 ubiquitin ligase, thereby programming it for proteasomal degradation (22, 23). Because C terminus mutations modulate SAMHD1 function, it is likely that Vpx binding to the C terminus will also affect SAMHD1 catalytic activity, for instance, by altering its tetrameric state. It will be interesting to see whether this domain can receive inputs from upstream viral and/or cellular regulators controlling cellular dNTP levels and translate them into SAMHD1 catalytic activity.

## Supplementary Material

Supplemental Data

## Footnotes

3 The abbreviations used are:

SIV: simian immunodeficiency virus
SAM: sterile α motif
AGS: Aicardi-Goutieres syndrome
PMA: phorbol 12-myristate 13-acetate
SEC: size exclusion chromatography
MALS: multiangle light scattering
HD-domain: histidine aspartate-motif containing domain.
