# Supplementary material for: Tetramerization of SAMHD1 Is Required for Biological Activity and Inhibition of HIV Infection
Source: J Biol Chem. 2013 Feb 20;288(15):10406–17. doi: 10.1074/jbc.M112.443796 (PMC3624423; doi:10.1074/jbc.M112.443796)
Supplement: Supplemental Data [file supp_288_15_10406__index.html]

Tetramerization of SAMHD1 Is Required for Biological Activity and Inhibition of HIV Infection — SAMHD1 Tetramer — Supplemental Data 

# Tetramerization of SAMHD1 Is Required for Biological Activity and Inhibition of HIV Infection

## Supplemental Data

**Files in this Data Supplement:**

- Supplemental Figures 1-2 &#x26; Legends (.pdf, 1.7 MB) - Supplemental Figures 1 &#x26; 2
