# Supplementary material for: Tetramerization of SAMHD1 Is Required for Biological Activity and Inhibition of HIV Infection
Source: J Biol Chem. 2013 Feb 20;288(15):10406–17. doi: 10.1074/jbc.M112.443796 (PMC3624423; doi:10.1074/jbc.M112.443796)
Supplement: Supplemental Data [file supp_M112.443796_jbc.M112.443796-1.pdf]

Supplemental Figure 1. dGTP induces SAMHD1 tetramerization. A. SAMHD1 (10 μM) was preincubated in the absence (**A**), or presence of 1 mM (**B**) or 4 mM (**C**) dGTP. The mixtures were injected into an analytical gel filtration column and separated at a flow rate of 0.8 mL/min. Elution profile (A280 nm) was recorded. The elution positions of tetramer and dimer/monomer peaks are indicated.

Supplemental Figure 2. SAMHD1 tetramer and dimer/monomer interconvert in a dGTP-dependent manner. A. A mixture of SAMHD1 (25 μM) and dGTP (45 μM) was injected to an analytical gel filtration column at a flow rate of 0.8 mL/min. Elution profiles (A254 nm, red trace, and A280 nm, blue trace) were recorded. Elution fractions corresponding to tetramer (1) and dimer/monomer (2) were collected, concentrated, and analyzed by SDS-PAGE and visualized by Coomassie Blue staining. The fraction containing tetramer (**B**) and dimer/monomer (**C**) from **A** were subjected to analytical gel filtration column chromatography. Elution profiles as revealed by fluorescence trace (excitation at 282 nm and emission at 313 nm) were recorded. **D.** The same fraction containing tetramers (1 in (**A**)) was mixed with 4 mM dGTP prior to gel filtration analysis. **E.** The fraction containing SAMHD1 dimer/monomer (2 in (**A**)) was incubated with 4 mM dGTP and then subjected to gel filtration analysis. **F.** The elution fraction containing tetramer shown as (1) in (**A)** was subjected to dNTPase assays a reaction buffer without or with dGTP (25 μM) added. The dA nucleoside product was quantified by HPLC. **G**. The elution fraction containing dimer/monomer fraction shown as (2) in (**A)** was subjected to dNTPase assays performed as in panel **F**.

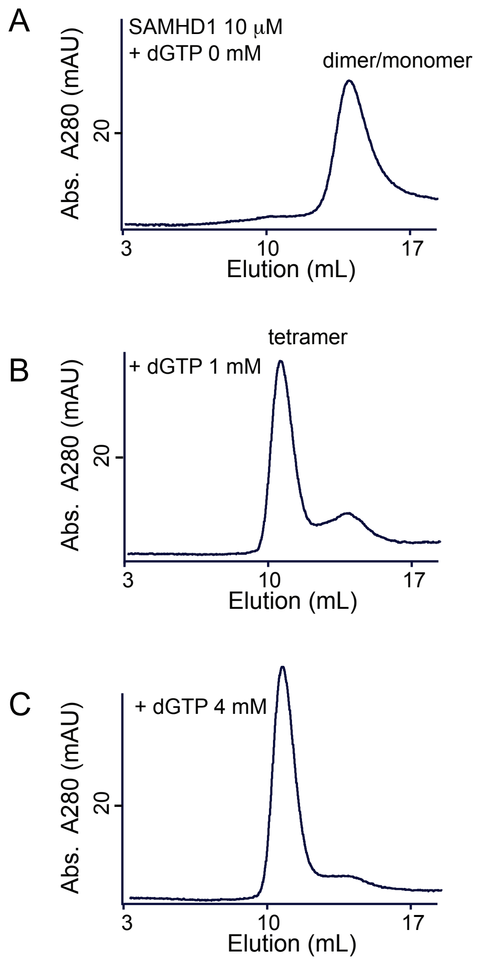

Supplemental Figure 1.

Yan et al.

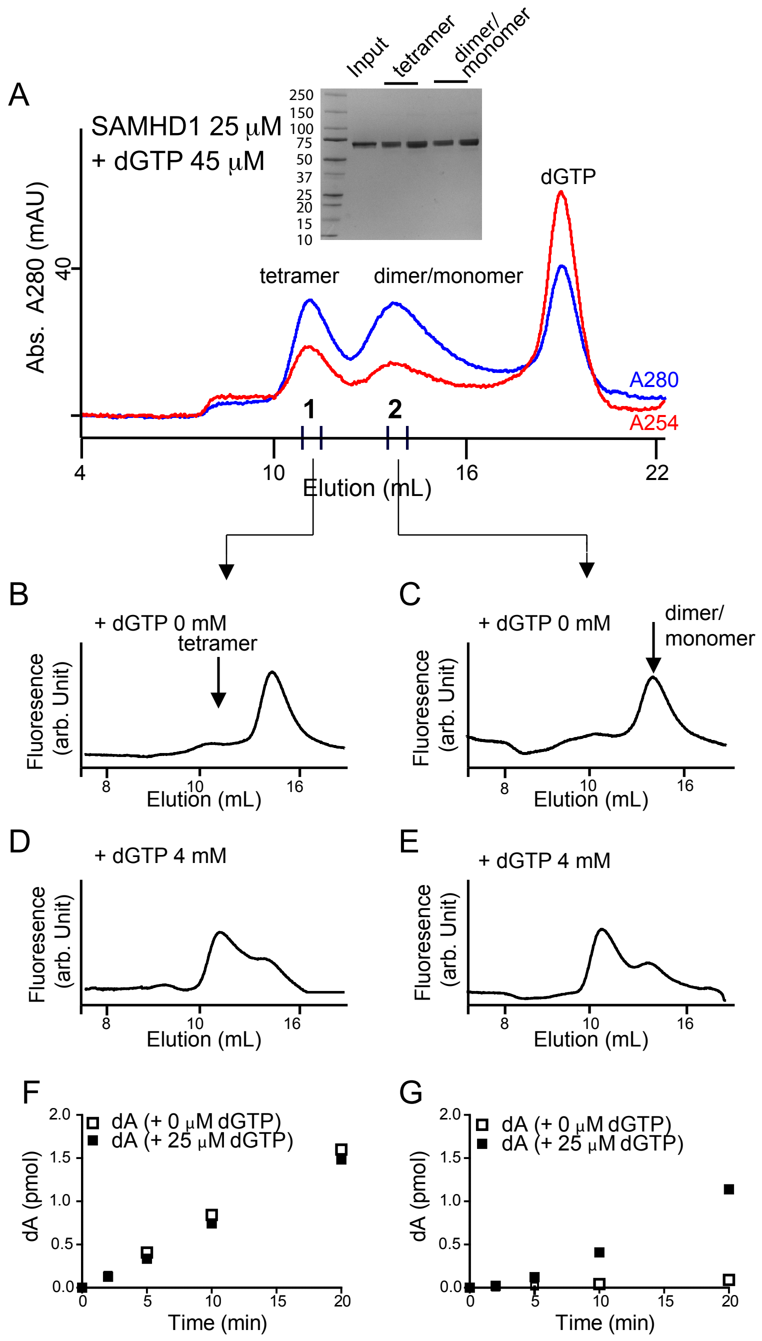

Supplemental Figure 2. Yan et al.
